# Sexual and reproductive health issues facing Southeast Asian beer promoters: a qualitative pilot study

**DOI:** 10.1186/1471-2458-10-389

**Published:** 2010-07-01

**Authors:** Gail C Webber, Denise L Spitzer

**Affiliations:** 1 Dept. of Family Medicine, University of Ottawa, 2450 Lancaster Rd., Units 11 and 12, Ottawa, Ontario, K1B 5N3, Canada; 2 Institute of Women's Studies, University of Ottawa, 30 Stewart Street, Ottawa, Ontario, K1N 6N5, Canada

## Abstract

**Background:**

In Southeast Asia, hundreds of thousands of young rural women migrate from their villages to the larger cities in search of work. Many find employment with beer companies or in the clubs where beer is sold, promoting the sale of beer. Previous research suggests these young migrants are in a highly vulnerable position. This paper will describe the findings of an October 2009 meeting to develop a research agenda on the sexual and reproductive health of beer promoters and a subsequent pilot study of focus groups with beer promoters to review this agenda.

**Methods:**

Participants of the research meeting representing beer promoters, academics, non-governmental organizations (NGOs), government and the beer industry from Cambodia, Thailand, Laos, and Vietnam collaborated in the development of three key research themes. The themes were verified in focus group discussions with beer promoters organized by local research partners in all four countries. The focus group participants were asked what they felt were the key sexual and reproductive health issues facing them in a non-directive and unstructured manner, and then asked to comment more specifically on the research priorities developed at the meeting. The focus groups were recorded digitally, transcribed, and translated into English. The data were analyzed by coding for common themes and then developing matrices to compare themes between groups.

**Results:**

The participants of the meeting identified three key research themes: occupational health (including harassment and violence, working conditions, and fair pay), gender and social norms (focusing on the impact of power relations between the genders on women's health), and reproductive health (knowledge and access to reproductive health care services). The participants in the focus groups in all four countries agreed that these were key priorities for them, though the emphasis on the most important issues varied between groups of women. Sexual harassment in the workplace and challenges in accessing reproductive health care services because of the barriers of cost, shyness, and stigmatizing attitudes of health care providers were common problems for many of the women.

**Conclusions:**

There is a need for regional research and programming for beer promotion women in Southeast Asia focusing on the three research themes of occupational health, gender norms and reproductive health. Such research and programs could provide important benefits for many beer promotion women who currently face significant risks to their sexual and reproductive health.

## Background and Methods

The Southeast Asian countries of Cambodia, Laos, Thailand and Vietnam have different histories and cultures, however, many of the region's residents share the common experience of migration within the country in search of better economic opportunities. In Cambodia alone, about one third of the population has migrated within the country [1] - often for economic reasons [2]. Hundreds of thousands of young rural women from these four Southeast Asian countries migrate from their rural villages to the larger cities in search of work to support themselves and their families. For example, as of 2008 about 350,000 people, mostly rural migrant women, worked in the garment factories of Cambodian cities [3].

A common occupation for migrant women in Southeast Asia is employment in the small restaurants, karaoke clubs, and other venues where alcohol is sold, promoting the sale of beer. While it is difficult to obtain accurate statistics on the number of women promoting beer, CARE Cambodia estimates that 4,000 women are employed as beer promoters in that country (personal communication, CARE Cambodia), and an International Labour Organization study determined about 1,550 beer promoters worked in the capital Phnom Penh in 2004 [4]. In Laos, only about 350 women officially work as beer promoters for the two main brands of beer, however, there are unofficial estimates of a further 8,000 to 10,000 women working in the bars, beer shops and nightclubs, earning commission on beer sales, in the capital Vientiane alone (personal communication, CARE Laos). There are no available statistics on the number of beer promoters in Thailand or Vietnam however; their numbers are also thought to be significant; thus thousands of women, many of them rural-to-urban migrants, are employed as beer promoters throughout the region.

Despite the fact that beer promotion women can be found throughout Southeast Asia, there is little research into the factors impacting their sexual and reproductive health. In addition, there are no cross border comparative studies of these populations of women. To address this gap, we organized a meeting to develop a regional research agenda on the sexual and reproductive health of beer promoters. This was followed by a pilot study with beer promoters in Cambodia, Laos, Thailand and Vietnam to assess the research priorities developed at the meeting. The research question addressed by the pilot study was: What are the key issues that affect the sexual and reproductive health of beer promoters, and how do these issues compare with the research agenda themes developed at the meeting? This paper will describe the results of the meeting and pilot study with beer promoters in these four countries.

Compared with their non-migrant counterparts, many migrant women are known to be at higher risk for sexual and reproductive health problems [5,6], including HIV and AIDS [7]. This is due to dual discrimination against both migrants and women that may develop into difficult social situations, including violence or threats of violence, and as a result may constrain access to sexual and reproductive health care services. Migrant women who work as beer promoters are no exception to this trend: the working conditions of the beer promotion industry are such that many of these young migrants are in a vulnerable position. A CARE Cambodia study [8] of 640 beer promoters conducted in Phnom Penh and provincial towns across Cambodia found that over 94% experienced unwanted sexual touching in the workplace at least a few times per month - almost 24% stated that it occurred nightly - while one third of the informants had experienced a coerced sexual act. Some beer promoters, however, move into commercial sex work, to supplement their incomes, in order to support their families. In addition, beer promoters who work on a commission only basis are often encouraged to consume alcohol on the job, which further increases their vulnerability to sexual risks. A study of beer promotion women in Battambang, Cambodia [9] noted that the HIV prevalence in this population was 26%, much higher than the general population, and 82% of the women reported a history of sex work.

One of the challenges of research with this population is that beer promoters are not a clearly defined group and the language used to describe these workers differs according to country and is informed by the organization of the industry in each nation. For example, in Laos, beer promoters who are employed by the beer company are differentiated from beer *sellers *who work for small restaurants and other establishments. Beer sellers have less status and job security as compared to beer promoters and are therefore more vulnerable to sexual advances and other workplace hazards. In the HIV surveillance data, however, sex workers in Laos are defined in such a way that they include those who engage even in infrequent commercial sex transactions to supplement their primary work selling food or beer [10]. In Thailand, beer promoters can be classified as indirect sex workers along with women who are employed in massage parlours [11]. Complicating this reality, observers have noted a shift in clients' preferences for indirect sex workers rather than brothel-based workers in Thailand [11]. Research conducted in Cambodia where beer promoters are classified as indirect sex workers revealed that about thirty percent of beer promoters had been paid for sex [12]. In Vietnam, sex worker statistics include both street sex workers and karaoke-based sex workers [13], thus there is an overlap with the beer promoter population in this country as well. Like street sex workers, karaoke-based sex workers in Vietnam demonstrate inconsistent condom use, with clients and particularly with regular partners, putting them at risk for HIV [13]. Thus, across Southeast Asia, the lack of clarity of the category of beer promoters in surveillance data obscures the risks for this population of women; while there is no doubt that many beer promoters supplement their income with sex work, it does not appear to be universally the case (as the data in Cambodia demonstrate [12]), nor is it possible to determine their HIV risks from the currently available data.

The HIV/AIDS epidemic has been prioritized as an important health issue to address by the governments of these Southeast Asian countries. In Cambodia, the national prevalence has fallen from 2% in 1998 to an estimated 0.9% among adults (aged 15-49) in 2006. The decline in the rate of new HIV infections is likely the result of government prevention programs [14]. Unfortunately, HIV prevalence remains higher amongst Cambodian beer promoters [8], 15% of whom were HIV positive in 2002 [12,15]. Importantly, beer promoters were less consistent in their use of condoms as compared to brothel-based sex workers, although use varied geographically [15]. The Ministry of Health in Cambodia has identified access to quality reproductive health information and services and HIV prevention as key priorities in their strategic plan [16].

Like Cambodia, Thailand has been able to achieve a decline in HIV prevalence through government prevention programs; however, a substantial proportion of new HIV infections are occurring in higher risk populations, including those working in the sex industry outside the brothel setting [14]. Similarly, Vietnam has a concentrated HIV epidemic among high-risk, mobile populations, including sex workers and migrants [17]. The HIV prevalence is continuing to grow in Vietnam, with an estimated adult prevalence rate of 0.5% in 2005 [14]. The government has put an emphasis on HIV prevention for the next decade in their national strategy [18]. In addition, a 2008 government circular on migration and HIV prevention highlights the importance of integrating reproductive health and other HIV prevention activities [19]. The extent to which beer promoters in Vietnam are involved in sex work or come to their trade through economic migration requires further study in Vietnam.

The limited data on the HIV epidemic in Laos suggest that while HIV prevalence remains low - as of 2001, the prevalence rate was 0.05% amongst 15 to 49 year olds [20] - there are worrisome signs. In a 2001 HIV and sexually transmitted infection periodic prevalence survey, almost 50% of "service women" (defined as women who worked in small drink shops, nightclubs or guest houses and had direct contact with patrons) were found to be infected with chlamydia and/or gonorrhea, two sexually transmitted infections [21]. Research and subsequent programming with beer promoters in Laos is particularly important as there is an opportunity to prevent the HIV epidemic from reaching the prevalence rates found in neighbouring Cambodia and Vietnam.

### Research Agenda Meeting

On October 5 to 7, 2009, we hosted a three-day meeting in Phnom Penh, Cambodia to develop a research agenda on the sexual and reproductive health issues facing beer promoters in Southeast Asia. Meeting participants included Cambodian, Laotian, Thai and Vietnamese representatives from government (e.g. Ministry of Women's Affairs, Police, Public Health), civil society (non-governmental organizations working with beer promoters), academics, the beer industry and beer promoters themselves. Prior to the meeting, we commissioned reports prepared by our research partner in each country that examined the current state of knowledge on the health and well-being of beer promoters in each locale.

The objectives of this meeting were to: 1. Define the reproductive health issues faced by women who promote beer in Southeast Asia and to explore similarities and differences across different populations; 2. Develop appropriate research questions for future research which will aid in the sexual and reproductive health needs assessment and the design of future interventions for this population; 3. Discuss and develop research methodologies and designs to address these research questions; 4. Identify potential research collaborators to participate in the research across different sectors (community, academic, civil society, governments, and industry); 5. Build networks across different sectors and across borders in order to build research capacity; 6. Identify sources of funding for this research both locally and internationally; and to, 7. Agree on the key content of a research proposal, in order to seek funding for future research. The key research themes developed from this meeting were used in the pilot study described below.

### Pilot Research Project

After the three-day research agenda meeting in Phnom Penh, the authors collaborated with their partners to hold a pilot study comprised of focus groups with beer promoters in Cambodia, Laos, Thailand and Vietnam. The objectives of this pilot study were to review the research priorities developed at the research meeting with the beer promotion women. Prior to data collection we sought and obtained ethics approval for this research from the Ottawa Hospital Research Ethics Board in Canada and - with the assistance of our research partners in each country - ethics committees in all four countries where the research was conducted. Table 1 outlines the ethics approval committee, location of the focus groups, and recruitment process in each country.

**Table 1:** Beer Promoter Pilot Study: Ethics Approval, Location and Recruitment Process

Country of Research	Ethics Approval	Location of Focus Groups	Recruitment Process
Cambodia	Ministry of Health, National Ethics Committee for Health Research, Cambodia	NGO Office, Phnom Penh, Cambodia	NGO recruitment of beer promotion women known through their programming

Thailand	Society for Anti-AIDS Danger and Life Quality Improvement, Bangkok, Thailand	Private room in beer promoter place of work, Bangkok, Thailand	Recruitment of beer promoters by local researchers in two very different local venues

Laos	Ministry of Public Health, University of Health Sciences, Vientiane, Laos	University classroom, Vientiane, Laos	Local beer company recruited beer promotion women

Vietnam	Ethics Committee for Human Research, Thaibinh Medical University, Vietnam	Private room in beer promoter place of work, Thaibinh, Vietnam	Recruitment of beer promoters by local researchers in local venues

The participants for the focus groups were recruited by our NGO partners in Cambodia, our academic partners in Thailand and Vietnam, and by the staff of a major beer company in Laos. A recruitment letter was used to explain the research to the women in the local language. The sampling framework was a convenience sample, i.e. we drew on beer promotion women who were both interested and available to participate in the research. Our two key selection criteria for informants were that: 1. they be at least age eighteen and thus be able to give consent, and 2. they have worked as a beer promoter for at least three months. Efforts were made to ensure that the women were not coerced to attend the focus groups. In particular, women were informed that their views would be kept confidential and that their identities would be concealed. In Laos, the researchers emphasized that they had no connection with the beer company employer and would not be informing the beer company of the women's views. In addition, the women were asked to keep the discussion confidential. Interpreters and research assistants signed a confidentiality agreement prior to the focus groups, while participants were asked to sign a consent form.

The focus groups were facilitated by the authors with the assistance of local interpreters and were conducted in a quiet location (see Table 1). In Vietnam, however, foreign researchers are prohibited from carrying out data collection themselves; therefore, the focus groups were conducted by local researchers after coaching by the authors. The focus groups were held in the local language and were all digitally recorded. The focus groups were designed to elicit discussion and the key sexual and reproductive health issues for beer promoters, and to confirm the research agenda developed in the meeting. The focus group guide of questions and explanations or probes is documented in Table 2. The primary research question addressed by the pilot study was: "What do you feel are the issues that affect the sexual and reproductive health of beer promoters?" After a general, unstructured and non-directive discussion about the important issues facing them, the women were informed about the research priorities developed at the meeting and asked to comment on them. In a more directive structured manner, the women were encouraged to specify what they thought were the most important sexual and reproductive health issues facing them, and how these were reflected in the research priorities developed at the meeting. The women were paid the equivalent of $9 to $10 US for participating in the focus groups.

**Table 2:** Focus Group Guide

Focus Group Question	Explanation of Question/Probes
1. What do you feel are the issues that affect the sexual and reproductive health of beer promoters?	We define sexual health as the freedom of the woman to have sex with whom she chooses and to keep safe from sexually transmitted infections. We define reproductive health as the freedom of the woman to choose if, when and with whom she chooses to have children with, and access to quality health care during her pregnancy and at the time of delivery as well as access to effective and acceptable family planning measures.

2. Of the issues discussed above, what would you consider are the three most important? Why?	The women were encouraged to name the most important issues of the ones they have raised thus far in the discussion.

3. What do you think about the importance of these issues raised at the meeting, and how do they compare with the issues we discussed in number 2?	We recently coordinated a meeting of government, civil society organizations, academics, beer industry and beer promoters in Cambodia. There were representatives from Cambodia, Laos, Thailand and Vietnam at the meeting. The following three issues were thought to be the most important for future research on beer promoters at this meeting: health issues in the workplace, gender and social norms, and reproductive health issues (examples were provided for each: see Table 3).

The digital recordings of the focus groups were transcribed by the local research assistants immediately after the focus groups were held. The transcriptions of the focus groups were then translated into English. The names of all the participants were changed to conceal their identity, and they were provided with a pseudonym. The Canadian researchers analyzed the data through multiple readings of the transcripts, coding for common themes by hand. This constant comparative method guided the analysis: similarities and differences between beer promotion women from each country were a focus of the analysis.

## Results

### Research Agenda Meeting

The October 2009 meeting to develop a research agenda on the sexual and reproductive health of beer promoters was attended by about 40 people from the four Southeast Asian countries, in addition to the two Canadian researchers. Participants of the meeting collaborated to develop the major research themes by gathering in inter-disciplinary country groups to brainstorm the important issues for beer promoters in their country, and then presenting their results to the larger group. From the four country presentations, the larger group developed three major research themes. These themes were further expanded by working groups on each theme. Importantly, these three themes were not intended to be exclusionary, but rather to capture all the possible issues facing beer promoters which may impact on their sexual and reproductive health, thus there is some overlap between the themes. The three research themes and the issues to be addressed within these themes are outlined in Table 3.

**Table 3:** Three Key Research Themes for Beer Promoter Research

Research Theme	Issues to be Addressed
Occupational Health	Violence, work contract, health insurance, exposure to smoke and noise, stress, sexual harassment, verbal harassment, alcohol consumption, workload, uniforms/high heeled shoes, impact on social and family life, access to health care, employer expectations of beauty, relationships between beer promoters and customers, discrimination, safety, infectious diseases, effects of using make-up, and salary and commission.

Gender and Social Norms	Negotiation skills, good/bad girl dichotomy, stigma, male behavior, uniforms, power relationship between men and women, gender ideology, status in family/bread winner, and family planning decisions.

Reproductive Health	Unsafe abortion, HIV risks, sexually transmitted infections, reproductive health counseling, maternal health, sensitivity, contraception use, family planning (range of family planning options and side effects/management), access to reproductive health services, knowledge/health education, precautions, HIV testing, emergency treatments, reproductive health policies, adolescent services, attitude of health care providers, cost and convenience of services, and male responsibility for reproductive health.

### Pilot Research Project

#### Research Participants

The focus groups with beer promoters from Cambodia, Laos, Thailand and Vietnam took place in the two-week period following the research agenda meeting. Seventy-one women participated in the eight focus groups (two focus groups per country). The number of participants was relatively evenly distributed between the four countries: Cambodia (19), Laos (18), Thailand (17), and Vietnam (17); however, the characteristics of the women participating in the focus groups varied somewhat between the groups. The Cambodian beer promoters were all poor migrant women who had come from the countryside to the city to support their families. They chose to work as beer promoters as they lacked education for other better-paying jobs. About half of the beer promoters in the first Thai focus group and almost all the beer promoters in the second Thai focus group were also migrant women from the countryside, often working to support their families as well as to provide themselves with better life options. Some of the Thai beer promoters in the first focus group (in a middle class establishment) were students supporting themselves. The majority of the Laotian beer promoters were from the capital, and several were also students supporting themselves through part-time work as beer promoters. Most of the Vietnamese beer promoters were from Thaibinh, a city of three million people, approximately 100 km from Hanoi, where the focus groups were held, although a few came from the surrounding countryside.

The following paragraphs describe the findings of the focus groups with beer promoters. Key themes the women raised in the groups were the economic factors that drove them to work as beer promoters, workplace harassment, other occupational risks, stigma, health care access, sexual and reproductive health conditions, and industry support.

#### Economic Factors

While all of the women working in beer promotion found work as beer promoters to earn an income, the rural-to-urban migrant women were often working particularly hard to provide financial support to their families in the countryside. For some of these women, every spare penny was sent back home. This Thai beer promoter described how her income was used:

Fifty percent goes to the family, the rest is for accommodation (renting room), meals, personal expenses. (Mee, Thailand FG1)

Other migrant women chose to send money home on a less regular basis, but still provided important support to their families in the countryside. A Vietnamese beer promoter described how she only sent funds for special purchases as she felt if she sent a little money each month her family would not be able to afford the larger essential items. The previous month, the money she sent allowed her family to obtain a new bathroom, for as she stated: "... you know that in the countryside the bathroom and toilet is very old and dirty." (Hue, Vietnam FG2)

Economic need was particularly acute for the women who were employed solely on commission. One of the Cambodian beer promoters described how some of these women were forced to supplement their income from promoting beer with sex work in order to provide for their families.

I think that sexual issues are the main challenge for beer promoters because some beer promoters are working with the commission-based system so when they cannot sell well then they go out with customers for some money to pay for room rental, utilities, and to support their parents in the homeland. Again, going out and sleeping with customers is not our intent; due to economics and high demand from within the family, finally we decide to sleep with customers. (Kimry, Cambodia FG2)

Chantou, another Cambodian beer promoter, provided an unfortunate example of the economic circumstances that compelled her to engage in part-time sex work in order to provide for her extended family. Chantou's younger sister had become pregnant before marriage. As the eldest child, Chantou felt it her responsibility to assist her sister in obtaining an abortion. The procedure cost $60 US - more money that she had in her possession. She related to us:

In order to get $60 for my sister, I had to sleep with two men within one night. My life is so difficult sometimes. I have problems and I don't have money. (Chantou, Cambodia FG1)

Economic factors were the drivers which brought women to work as beer promoters and which may force them to make difficult choices about engaging in sex work. Migrant women with family responsibilities were particularly vulnerable to economic pressures and thus may be more at risk of using sex work as a supplement to their income. Those women who lacked the security of a stable salary and worked on commission only were also more vulnerable to resorting to sex work to supplement their income. Chantou's story illustrates the desperation that some of these women face. Sex work was seen by them as their only option for meeting their personal and family obligations.

#### Workplace Harassment

Workplace harassment was a universal problem for the beer promoters in all four countries. The harassment varied from verbal abuse to unwanted touching and sexual propositions, to threats of severe violence. Not surprisingly, alcohol was often a major factor in the harassment. This Laotian beer promoter summarized the situation:

When the clients are drunk - very drunk, they try to touch our bodies and we can't do anything about that except look at their face. (Chanthone, Laos FG1)

Na, a Vietnamese beer promoter quoted below, described a memorable night when several men physically harassed her in her workplace and badly frightened her. Initially, she felt things were going well as they ordered and drank a large quantity of beer while they sang karaoke. It was her job to stay with them and continue to serve them. Unfortunately as more beer was consumed, the behaviour of the clients became increasingly unacceptable:

But after about one hour serving them, one of the men started flirting me with bad words and then he touched my thigh under my short skirt. I felt uncomfortable and tried to be serious and just poured beer for those men. But then, three men suddenly stood up and stood around me - they stood like a circle around me and pretended to be singing and used their hands to touch my breast, bottom and thigh. I was shocked and tried to use my words to stop them, but they pretended not to hear me and continued that. (Na, Vietnam FG2)

Na escaped by fleeing from the room. She confessed that it was an "unforgettable time" for her, but that she was more experienced now, and able to avoid those situations. Na's story was not unique for other beer promoters recalled being physically and sexually harassed in the workplace. While not common, the Cambodian beer promoters related stories of being drugged by clients, and receiving threats of severe violence. They experienced work as a beer promoter to be very risky at times.

During my first month as a beer promoter in one restaurant, there was one customer who ordered my brand and asked me to sit with him. He said all the time he met me he spent a lot of money and he said if I don't sit and drink with him, he will shoot me. I was very nervous and scared of him. (Jorani, Cambodia FG 1)

As the women were economically dependent on their work as beer promoters, they often tolerated the abuse, having few other options. With experience, sometimes they managed to avoid troublesome clients by moving to another part of the venue. Workplace harassment did not always originate with the clients, however. One Cambodian beer promoter related how the manager of the outlet where she was stationed to sell beer demanded sex from her, and threatened her with the loss of her job if she did not comply.

I had a conflict with the outlet manager. He asked me to sleep with him several times and he said he will pay me $10 US. He added that if I don't agree to sleep with him - he will fire me. He will report to my company to take me away from this place. (Mei Channy, Cambodia FG2)

Thus workplace harassment from clients, and occasionally from other staff working in the venues where beer is sold, was a common and serious problem for the beer promoters. Amongst the participating women, experiences of workplace harassment varied from comments and looks to unwanted touching and even threats of severe violence as noted above. Their need for income made them vulnerable to such abuse. Workplace harassment, while common, problematic, and at times severe, was not the only risk they faced on the job, however, for the beer promoters also reported other occupational risks.

#### Other Occupational Risks

In addition to varying degrees of harassment, these women cited other issues in the workplace environment that they felt impacted on their health, most notably loud noise and second-hand smoke. The beer promoters felt that exposure to pollutants put their health at risk.

I am also exposed to smoke during my duty. Some male clients just blow the smoke of cigarettes in my face on purpose causing me to have a headache. (An, Laos FG 2)

We have to bear smoke and noise in karaoke, it is difficult to breathe. At first I couldn't bear it: after working time, my body, clothes, hair all have cigarette smell. I just wanted to vomit. (Ngoc, Vietnam FG 2)

Beyond workplace noise and smoke, some of the women felt that the expectations for their physical appearance were problematic for their health. Beer promoters are expected to be physically attractive as this is thought to please the male clients and increase the sale of beer. A couple of women mentioned that they did not like to have to wear make-up. In order to improve their appearance, some beer promoters were also required to wear uniforms for their positions that for some women included high-heeled shoes.

You know we have to wear high-heel shoes, make-up for a long time. I usually get muscle pain and uglier skin on my face. (Nga, Vietnam FG2)

Standing for many hours gives me pain in my back. I also think that it is a result of wearing high-heeled shoes. (Bo, Laos FG 2)

Alcohol consumption was also a health concern for some of the beer promoters. This was particularly the case for the Cambodian beer promoters who worked on commission, as they faced pressure to drink with their clients, in order to encourage the increased consumption of the product they were promoting.

As a beer promoter we have to drink beer everyday with the clients, so it can destroy our stomachs. (Kolab, Cambodia FG 1)

This was not the case for all the women working as beer promoters elsewhere: in Laos and Vietnam the women participating in the focus groups reported that they were generally not allowed to drink with their clients.

Facilitator: "Have you ever been invited to drink beer with customers?"

Respondent: "Yes, sometimes, but we are not allowed to sit with customers so we just drink a little and then thank them because I still have work to do." (Van Anh, Vietnam FG 2)

Thus work as a beer promoter was viewed as having several risks to health. The most significant was the sexual harassment the women experienced, but noise and smoke, and the rigours of having to appear attractive all the time were also perceived as problematic. The over-consumption of alcohol was a concern for some of the Cambodian beer promoters.

#### Stigma

Beyond the occupational risks of harassment and the environmental problems of the workplace, the beer promoters in all four countries described experiencing stigma because of their work. Generally the stigma was related to the perceived association of beer promotion with sex work. The stigma was experienced at many levels: general society, male clients, family members and even health care providers could be the proponents of stigmatizing attitudes. This Vietnamese beer promoter described how her desire to be understood as a responsible young woman who was working to support herself was thwarted by society's views of her occupation. Her Thai colleague was also conscious that her society did not approve of her choice of employment.

It is social norms. I hate being looked at with bad eyes by other people because they think that I am a beer promoter and I am not a good girl and I have sex with customers. I much hope that they will understand my job. (Nga, Vietnam FG 2)

Thai people are also the same, the beer promoter women are looked down by the public as 'easy for having sex'. (Kanya, Thailand FG 1)

Some male clients also perceived the beer promoters as being available for sex. This was particularly obvious if they had the resources to pay for sexual services:

Men at the age of about 40 have money and like to ask us to come home to sleep with them. They see us as toys that they can play. They think that we are sex workers. (Laya, Laos FG 1)

Family members held stigmatizing beliefs about the work of beer promoters as well. These beliefs could lead to disagreements within the family, and motivated some beer promoters to hide the details of their jobs from their families. For example, the two beer promoters quoted below perceived significant pressure from their families to avoid the appearance of being a sex worker by promoting beer. In the first case Chenda disregarded her father's advice to leave her job, while in the second Ly deliberately concealed the nature of her job from her family.

My community at my homeland discriminates against beer promoters and one of my family members doesn't like my job at all. My aunt spread the rumor in the community that I work in Phnom Penh as a prostitute and my father feels bad about that. I keep discussing with my father about my job and I told him that the important thing is we have meals three times a day. My father heard the rumor from my aunt like this and he asked me to return back to the homeland and he can do the farm work to support me. He is angry with me as I don't follow his advice... (Chenda, Cambodia FG 1)

I don't dare to tell my parents that I am beer promoter as they are living in countryside and to them, beer promoter means we have sex with men and get money from that. I just tell them I work in small restaurants as a waitress. (Ly, Vietnam FG 2)

For others, the economic needs of the family surpassed the stigma of the job, and family members did not comment on any beliefs they may have held. This was the case for this Thai beer promoter:

I informed my family [about my job], they don't say anything because I am the one taking care of my family because my family are farmers, and we have less money. (Mee, Thai FG2)

The beer promoters also experienced stigmatizing attitudes from their health care providers. Some chose not to disclose the nature of their work to the health care provider in order to avoid such attitudes.

Facilitator: "When you go to access health care - when you go to see doctor or nurse in the health centre - do you ever feel they treat you badly because you're beer promoters?"

Phirom: "They did not treat us the same as the others... They speak to us with rude words." (Cambodia FG 2)

Facilitator: "Are you afraid that doctors know that you are a beer promoter?

Nguyet: No, I often do not tell them that I am a beer promoter, I just tell them a normal job like staff of an office or waitress or I sell clothes, etc."

Facilitator: "Oh, why? Will doctors treat you different if they know that you are a beer promoter?"

Nguyet: "No, but I do not want them to look at me a different way. I often go to private clinics to have my health examined and I just pay them money and I will be treated as others. Nothing different." (Vietnam FG 1)

Stigmatizing attitudes towards the beer promoters were a common theme for all the women. They felt the stigma from the society around them as well as from their clients, their family, and their health care providers. The attitudes of family members and health care providers led them to sometimes conceal the nature of their occupation. Health care provider stigma has a direct impact on the access of beer promoters to quality health care. If they feel they are going to be judged as sex workers or "bad girls" then women are less willing to present to clinics or hospitals for their sexual and reproductive health concerns. It is possible that the beer promoters also perceive differences between private and public health care providers as the quote above suggests, for in the private system people tend to pay more, and thus they may feel entitled to hide their occupation as they have purchased the service. The stigma beer promoters experience from health care providers is only one of the issues limiting health care access, however.

#### Health Care Access

While many women stated that health care services were available in the cities they were working, they did not always have easy access to them. In addition to health care provider stigma, lack of time, cost, availability, and shyness were other reasons for avoiding health care services for some women. For example Ma, a Thai beer promoter, noted that she could not afford to take time off from work to go to a clinic, thus she self-medicated by accessing her local pharmacy.

I don't want to see doctors because I get a daily wage. If I stop working one day, I will not get paid. I like to buy drugs from the pharmacy store. (Ma, Thailand FG 2)

The lack of financial resources for beer promoters limited their access to certain services. Sophea, a Cambodian beer promoter, noted that she and her colleagues had to wait longer for health care services as they were not in the priority group for appointments:

To me, I feel it is difficult as the health services of some NGO [non-governmental organizations] are separated into different categories including appointment services for clients who have money and people like us. We don't have money so we have to wait and sometimes treatment could not be done, then we return back home. (Sophea, Cambodia FG2).

Access to abortion services was an issue raised by the Cambodian beer promoters in particular.

It is not easy at all to get abortion services. Some NGO [non-governmental organization] clinics don't provide abortion services so we decide to buy medicine from private clinics by ourselves. Some NGO clinic scolded that here is not a place for abortion. It is a place for pregnancy care. (Phirom, Cambodia FG 2)

The beer promoters from Laos often relied on their mother's advice for health problems. They were shy to discuss their health issues with a physician, and were also concerned about the cost of health care services.

My mum is the first person with whom I consult my health issue. If I feel that the problem is not a serious, I will self treat by taking some medicine bought from a drug store. The doctors will diagnose the problem if I go to the hospital and it can be expensive. Also, I feel shy to go to the hospital. (Lian, Laos FG 2)

Thus, the limitations on access to health care services for beer promoters have an important impact on their sexual and reproductive health. In addition to health care provider stigma, lack of time, cost, and shyness are all contributing factors.

#### Sexual and Reproductive Health Conditions

The focus group participants reported on several sexual and reproductive health symptoms that gave them concern. Vaginal discharge was a common symptom and several of the beer promoters commented that this was a problem for them. While it was not clear that this symptom always equated with a sexually transmitted infection, for women who chose to have sex with their clients in order to generate more income to support themselves and their families, this was clearly a risk. Getting clients to use condoms was not always possible, thus the lack of safe sex and the risk of sexually transmitted infections was a definite concern for these beer promoters.

We don't want to say that we never have sex. We sometimes have sex, but some people do not have any issues about their reproductive health. Some others have serious discharge from their vaginas. (Jun, Laos FG 1)

The issue is like this: when we went out with a customer and then when we sleep together and the customer did not use the condom, that it is a problem for us. If the customer did not use the condom like this, it causes us to have vaginal discharge and also an irritated womb. Some male clients do not want to use condoms with us because they think that using condom is not natural. (Botum, Cambodia FG 1)

Other reproductive health conditions the beer promoters expressed concerns about included AIDS and cervical cancer. A few beer promoters raised stories they knew about colleagues who had gotten HIV/AIDS from a client. The Thai beer promoters appeared to be more sensitized to the risk of cervical cancer than the women from the other countries:

Facilitator: "Do you know someone having health problems after they go out with the clients?"

Mee: "Two of my friends got HIV infection, having papules, mucous and died."

Ta: "I have many friends who got cervical cancer." (Thailand, FG 2)

Thus sexual and reproductive health conditions were a concern for many of the beer promoters. Vaginal discharge, safe sex, HIV/AIDS and cervical cancer were all raised as issues. For those who engaged in sex work, getting clients to use condoms was a challenge. There is clearly a need to address concerns about these sexual and reproductive health conditions in this population.

#### Industry Support

The beer promoters in the focus groups in Laos were unique amongst the research participants in that they all worked for the same major national beer company. These beer promoters generally spoke with pride about their company as they felt the company had taken the initiative to develop policies that would protect their safety.

[Our] Company is responsible for the life of every [Our] beer promotion woman. I want to mention about our competitor, [The Other] Beer Company. Some of the [The Other Company] beer promoters live far away from the company or beer shops so they choose to come to work on their own. They dress beautifully or sexily covered with a jacket. They also wear uniforms at work. Sometimes those women don't come home. They go with their clients so they are not safe like [Our] beer promotion women. (Lai, Laos FG2)

We are not allowed to sit down with clients, eat or drink with them. Also, we are not allowed to come to work or go home after work alone - by ourselves. We have to be in the company's bus. Importantly, we set rules for ourselves to follow to protect ourselves from being harmed at/from work. (Wei, Laos FG 2)

These Laotian beer promoters recognized the benefit of working for a supportive company that took some measures to protect their safety. In particular, company- provided transportation to and from work limited the opportunities for clients to harass them. Rules preventing eating and drinking with clients also helped protect the beer promoters from unwanted attention from clients. These are just a few examples of how relatively simple measures such as prohibiting drinking with clients and the provision of transportation services has a large positive impact for individual beer promoters and indicates that there is an important role for the beer industry to take a greater interest in the safety of its employees.

#### Reviewing the Research Themes

At the end of each focus group, the facilitators reviewed the conclusions of the research meeting with the research participants. The beer promoters were asked if they felt the three major themes derived from the meeting (occupational health issues, gender and social norms, and reproductive health issues) reflected their discussion. The responses varied by focus groups. Generally, the women agreed with these three themes, however, the emphasis varied between the groups. The Cambodian beer promoters emphasized the issue of violence and harassment and health care provider attitudes, while the Thai women raised the environmental issues of noise and smoking, as well as stigma. One Thai group emphasized that they wanted more reproductive health knowledge. The Laotian beer promoters were more concerned about gender and social norms than reproductive health as they felt their access to health care services was adequate. Similarly, the Vietnamese women stressed working environment and social norms over reproductive health, as they felt their access to health care was also acceptable. Interestingly, while the Laotian and Vietnamese focus group participants felt that they had sufficient access to reproductive health care, their stories during the focus groups suggested that that cost, shyness and health care provider attitudes (stigma) may be limiting factors for their access.

## Discussion

This research meeting and pilot study on the sexual and reproductive health issues facing beer promoters in four Asian countries confirmed several of the findings in previous research. Unwanted touching and sexual harassment, as demonstrated in the CARE Cambodia study [7], was a common problem for all the beer promoters. Sex work and risks of sexually transmitted infections, including HIV/AIDS were discussed more by the Cambodian beer promoters, though women from the other countries were certainly aware of beer promoters who engaged in sex work in their countries. Further quantitative studies would be required to see how the rates of sex work and HIV/AIDS in beer promoters across Southeast Asia compare with those of the Battambang Cambodia study [8]. In addition to confirming the previous research findings, the research meeting and pilot study with beer promoters developed a broad research agenda. The three major themes of occupational health issues, gender and social norms and reproductive health issues as described in Table 3 provide a guide to future research on the sexual and reproductive health issues facing beer promoters in Asia.

The impact of the current global economic crisis on the labour market and the financial wherewithal of households in Southeast Asia suggest that an increasing number of young women will be turning to beer promotion work to sustain themselves and their families [8,12,22]. Inflating the number of beer promoters enhances competition and drives down wages creating a situation that is particularly problematic for those working on commission. Resultantly more women may resort to sex work to supplement their incomes. As indicated, part-time, non-brothel based sex workers may be increasingly vulnerable to STIs and HIV infection due to inconsistent condom use; therefore, work with this population is critical at this time to ensure that reproductive health programs and policies are adequately addressed and resourced [15].

There are several limitations to this research. As this was a pilot study, the numbers of beer promoters involved in the study and the time spent with each group was limited. In addition, a variety of recruitment strategies were used, thus the population of beer promoters recruited to participate in the study differed between countries. It is therefore difficult to make conclusions about country differences in the beer promotion industry in Southeast Asia based on this small pilot study. This study was strengthened by the initial research meeting to define the issues, with the involvement of a number of different stakeholders from each country (e.g. beer promoters, non-governmental organization staff, government staff, academics, and beer industry). In addition, the variety of recruitment strategies (through non-governmental organizations, academic partners recruiting in different venues, and beer company assistance with recruiting) provided a diverse group of research participants. While this diversity made between-country comparisons unfeasible, the confirmation of the three themes by all the focus groups was a means to triangulate the data, affirming the importance of the research themes in all regions by a diverse group of women.

There are several challenges for future research with beer promoters in Southeast Asia. Identification and inclusion of the most vulnerable groups of beer promoters, migrant women, may require specific recruitment strategies: collaboration with non-governmental organizations running programs with this population (as was done in Cambodia in this study) is likely the most efficient route; however, reliance on a partner can have an impact on the sample as these individuals are likely to take up the values and perspectives of the organization, potentially creating more attitudinal homogeneity than is reflected in the larger populace. Using snowball techniques beginning from different access points in the target community is vital to increase the variety of women sampled and may help us avoid this potential bias. Comparison between countries on certain issues such as reproductive health care access will require a deeper understanding of the health care systems of each country in both the public and private sectors. Coordinating a multi-site research project with four different working languages is also a significant challenge, however, this pilot study is evidence that it can be done through collaborative partnerships within each country.

## Conclusions

There are several conclusions that can be made from this research. The success of the research meeting and pilot study of beer promoters demonstrates that it is possible to organize a meeting with a diverse group of stakeholders and produce a relevant and useful research agenda. Beer promotion women in Cambodia, Laos, Thailand and Vietnam agreed that occupational health, gender and social norms, and reproductive health issues were all important issues that impact on their sexual and reproductive health. The emphasis on different themes varied depending on the population of women: preliminary observations suggest that there may be differences amongst some issues by country, social class, and health care system, but further research is required to study this in more depth. The Laotian beer promoters illustrated that beer industry support can have a large impact on protecting women from some of the vulnerabilities they experience (e.g. through transportation home from work, supportive policies, etc.). Examination of the beer industry's role in protecting the sexual and reproductive health of their beer promoters would be an interesting area for comparative research. Future research with beer promoters will need to carefully define and select the population if research with migrant populations is the goal. Collaboration with non-governmental organizations working with this population may be helpful to accessing migrant women.

In summary, beer promoters across Southeast Asia live and work in conditions that put their health at risk. The factors impacting on their sexual and reproductive health are broad and involve issues varying from working conditions, to access to reproductive health care knowledge and services, to society norms of how women are treated. While there are differences between populations, there are also some significant overlaps in experience between beer promoters in these four countries. In addition, similar programs for beer promoters are being developed by non-governmental organizations independently in these countries. There is therefore a need for further regional research and program development for beer promoters on occupational health, gender norms and reproductive health issues. A regional approach to research and program development could strengthen the research findings, improve program delivery, and have a positive impact on policies affecting beer promotion women, and thus lead to improved sexual and reproductive health for this vulnerable population.

## Competing interests

The authors declare that they have no competing interests.

## Authors' contributions

GW conceived of the research meeting, developed the funding proposal, analyzed the data and wrote the initial draft of the paper. DS conceived of the pilot study and did substantive editing of the paper. GW and DS both participated in the meeting and data collection. Both authors approved the final manuscript.

## Authors' information

Gail Webber is a community family physician and Assistant Professor of Family Medicine at the University of Ottawa, Canada. She has a doctorate in Population Health, and an interest in global health, women's health issues and HIV prevention. Denise Spitzer is the Canada Research Chair in Gender, Migration and Health at the University of Ottawa. She is also an Associate Professor in the Institute of Women's Studies and a Principal Scientist in the Institute of Population Health.

## Pre-publication history

The pre-publication history for this paper can be accessed here:

http://www.biomedcentral.com/1471-2458/10/389/prepub
